# Supplementary material for: Isolation and Identification of Microvirga thermotolerans HR1, a Novel Thermo-Tolerant Bacterium, and Comparative Genomics among Microvirga Species
Source: Microorganisms. 2020 Jan 10;8(1):101. doi: 10.3390/microorganisms8010101 (PMC7022394; doi:10.3390/microorganisms8010101)
Supplement: Supplementary file 1 [file microorganisms-08-00101-s001.pdf]

Isolation and Identification of *Microvirga thermotolerans* HR1, a Novel Thermo-Tolerant Bacterium, and Comparative Genomics among *Microvirga* Species

Jiang Li [1,2], Ruyu Gao [2], Yun Chen [2], Dong Xue [2], Jiahui Han[2], Jin Wang [1,2], Qilin Dai [1], Min Lin [2], Xiubin Ke [2,*] and Wei Zhang [2,*]

1 School of Life Science and Engineering, Southwest University of Science and Technology, Mianyang, Sichuan 621010, China;

2 Biotechnology Research Institute, Chinese Academy of Agricultural Sciences, Beijing 100081, China;

* Correspondence: kexiubin@caas.cn (X.K.); zhangwei01@caas.cn (W.Z.)

Supplementary Materials:

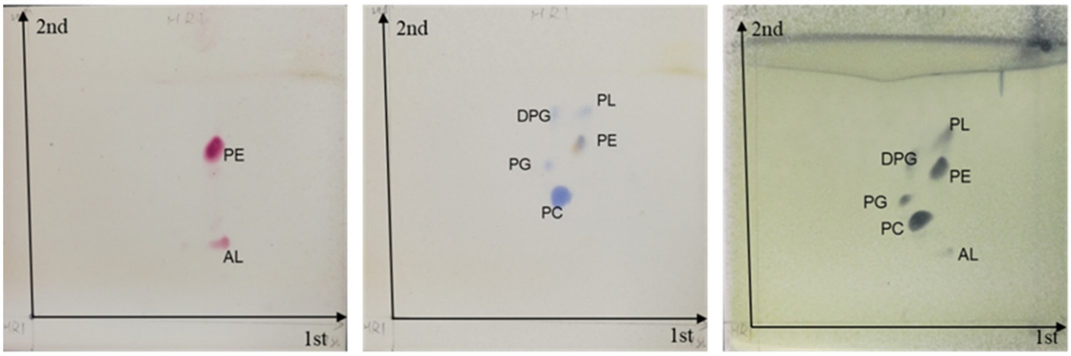

(**a**) (**b**) (**c**)

Figure S1. Two-dimensional TLC of polar lipids of strain HR1. Chloroform/methanol/water (65:25:4, *v*/*v*/*v*) was used in the first direction, followed by chloroform/aceticacid/methanol/water (80:18:12:5, *v*/*v*/*v*/*v*) in the second direction. The plate was sprayed with 5% ethanolic molybdophosphoric acid. The color agent of (**a**), (**b**), (**c**) was ninhydrine, molybdenum blue, and molybdophosphate, respectively. Diphosphatidylglycerol (DPG); phosphatidylethanolamine (PE); phosphatidylglycerol (PG); phosphatidylcholine (PC); phospholipids (PL); aminolipid (AL).

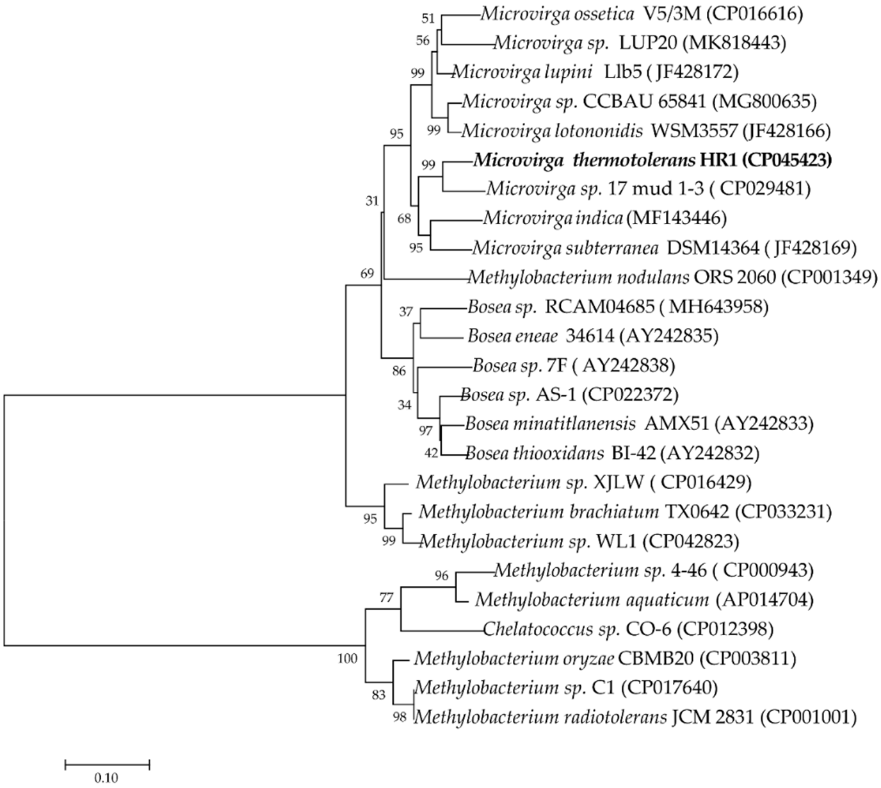

Figure S2. Phylogenetic tree based on the *rpoB* sequence. This tree was constructed by the neighbor-joining method, and showed the phylogenetic relationship between strain HR1 and closely related species.

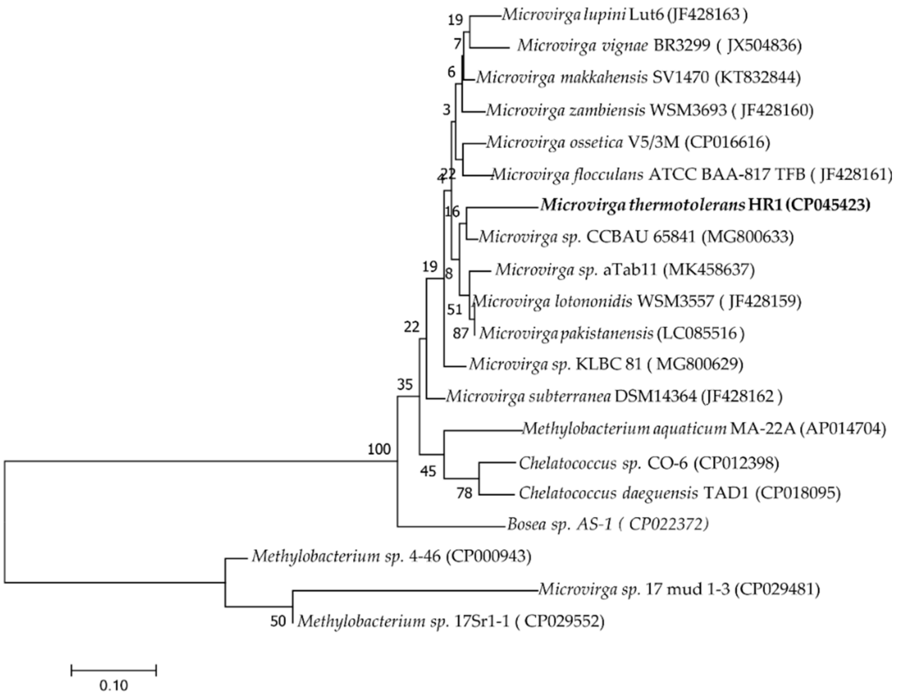

Figure S3. Phylogenetic tree based on the *gyrB* sequence**.** This tree was constructed by the neighbor-joining method, and showed the phylogenetic relationship between strain HR1 and closely related species.

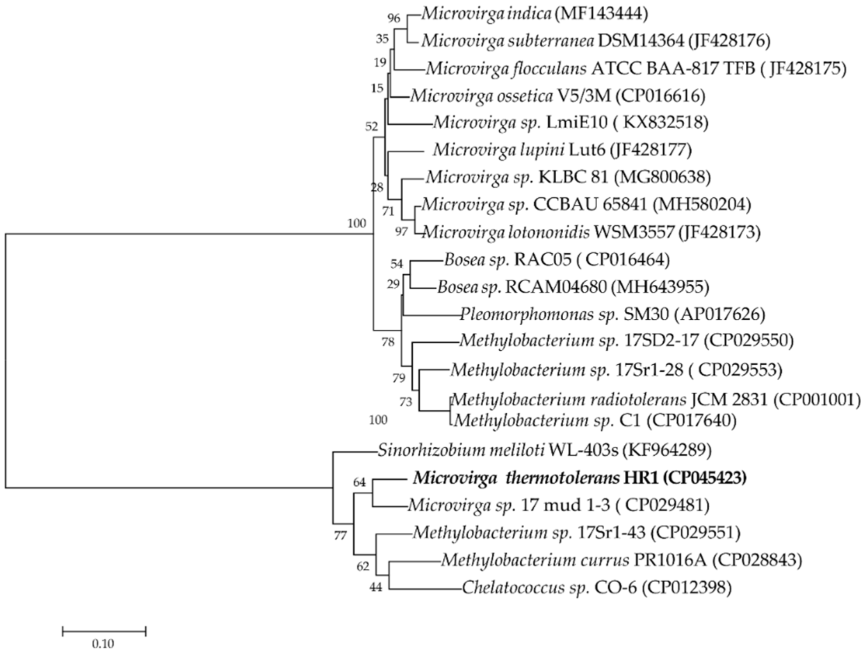

Figure S4. Phylogenetic tree based on the *recA* sequence. This tree was constructed by the neighbor-joining method, and showed the phylogenetic relationship between strain HR1 and closely related species.

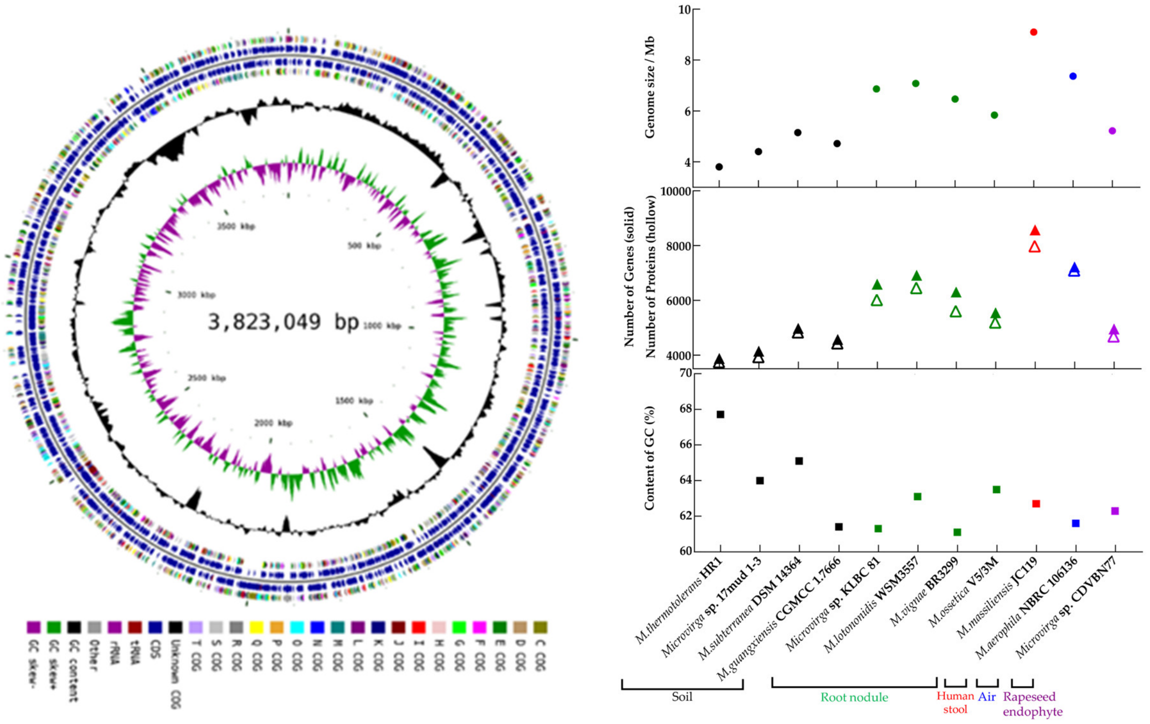

(**a**) (**b**)

Figure S5. (**a**) Circular genome map and circular representation of the *Microvirga thermotolerans* HR1 3.82 Mb chromosome. (**b**) Scatter plot illustrating whole-genome sizes, average percent GC content, and numbers of predicted genes and encoded putative proteins.

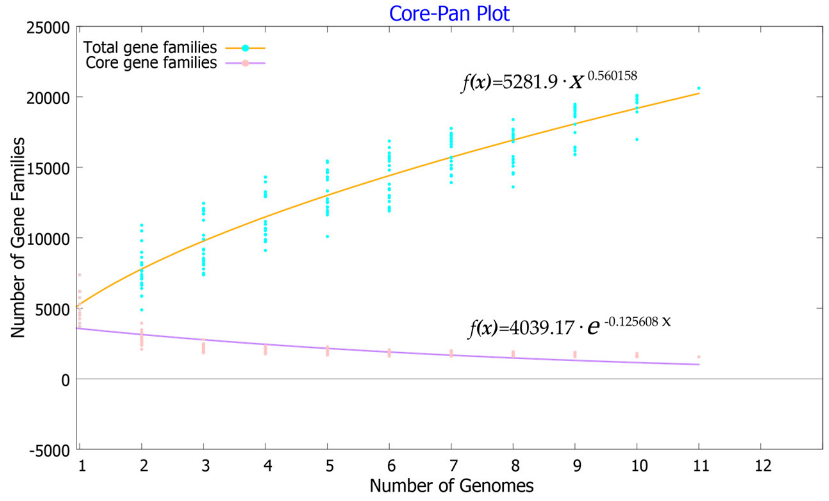

(**a**)

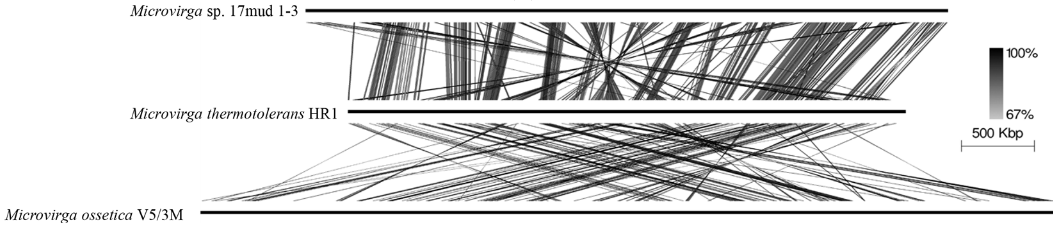

(**b**)

Figure S6. Mathematical modeling and linear comparison of *Microvirga.* (a) Mathematical modeling of the pan genome and core genome of *Microvirga.* (**b**) Linear comparison of the complete genomes in the *Microvirga* strains.

Table S1. The genes coding protein relative to nitrogen fixation and nodules formation in *Microvirga* genomes.

Microvirga Vignae BR3299		*Microvirga Guangxiensis* CGMCC1.7666	
Protein ID	annotation	Protein ID	annotation
KLK89555.1	nitrogen fixation protein NifW	SCX86875.1	NifU homolog involved in Fe-S cluster formation
KLK90284.1	nitrogen fixation protein NifX	SCZ11145.1	Radical SAM superfamily enzyme, MoaA/NifB/PqqE/SkfB family
KLK90285.1	nitrogenase iron-molybdenum cofactor biosynthesis protein NifN	SCZ11151.1	NifU homolog involved in Fe-S cluster formation
KLK90286.1	nitrogenase iron-molybdenum cofactor biosynthesis protein NifE	SCY97576.1	nitrogen regulatory protein P-II family
KLK90561.1	iron-molybdenum cofactor biosynthesis protein NifQ	SCY82617.1	NtrC family, nitrogen regulation sensor histidine kinase GlnL
KLK93004.1	nifZ protein	SCY82600.1	NtrC family, nitrogen regulation response regulator GlnG
KLK93006.1	nitrogen fixation protein NifB	SCY82575.1	NtrC family, nitrogen regulation sensor histidine kinase NtrY
KLK93698.1	nitrogen fixation protein NifU	SCY82559.1	NtrC family, nitrogen regulation response regulator NtrX
KLK93996.1	nitrogen regulatory protein	SCY79163.1	nitrogen regulatory protein P-II family
KLK91767.1	nitrogen fixation protein FixI	SCY72062.1	Nitrogen fixation protein FixH
KLK91765.1	nitrogen fixation protein FixL	SCY71901.1	Nitrogen fixation regulation protein
KLK90288.1	nitrogenase molybdenum-iron protein subunit alpha	SCY57825.1	PTS system, nitrogen regulatory IIA component
KLK90287.1	nitrogenase molybdenum-iron protein subunit beta	SCY48327.1	PTS IIA-like nitrogen-regulatory protein PtsN
KLK89635.1	nodulation protein NodU		
KLK89637.1	nodulation protein NodJ		
KLK89639.1	nodulation protein NodZ		
KLK89789.1	Nodulation protein W		
KLK91190.1	nodulation protein NolV		
KLK91191.1	nodulation protein NolT		
KLK91850.1	Nodulation protein W		
KLK92820.1	nodulation protein NolW		
Microvirga Lutononidis WSM3557		*Microvirga* Sp. KLBC 81	
Protein ID	annotation	Protein ID	annotation
EIM24386.1	NifQ	PVE24750.1	NifU family protein
EIM27506.1	iron-sulfur cluster biosynthesis protein, NifU-like protein	PVE21492.1	nitrogen fixation protein NifQ
EIM29761.1	putative TIM-barrel protein, nifR3 family	PVE21501.1	nitrogenase iron-molybdenum cofactor
EIM30571.1	iron-sulfur cluster biosynthesis protein, NifU-like protein	PVE21502.1	nitrogenase iron-molybdenum cofactor
EIM30714.1	nitrogenase cofactor biosynthesis protein NifB	PVE21503.1	nitrogen fixation protein NifX
EIM30716.1	NifZ domain-containing protein	PVE21504.1	NifX-associated nitrogen fixation prot
EIM30732.1	nitrogen fixation protein NifX	PVE21183.1	nitrogen fixation protein NifZ

Table S1. *Cont.*

EIM30733.1	nitrogenase molybdenum-iron cofactor biosynthesis protein NifN	PVE20977.1	cysteine desulfurase NifS
EIM30734.1	nitrogenase molybdenum-iron cofactor biosynthesis protein NifE	PVE21515.1	nitrogenase molybdenum-iron protein
EIM31020.1	Nitrogen fixation protein NifW	PVE21500.1	nitrogenase molybdenum-iron protein
EIM30737.1	nitrogenase iron protein	PVE22099.1	nitrogen fixation protein FixH
EIM30736.1	nitrogenase molybdenum-iron protein alpha chain	PVE21676.1	nodulation protein NOLX
EIM30735.1	nitrogenase molybdenum-iron protein beta chain	PVE21685.1	nodulation protein NolW
EIM30717.1	putative nitrogen fixation protein FixT	PVE20930.1	nodulation protein NolU
EIM29760.1	signal transduction histidine kinase, nitrogen specific	PVE20939.1	nodulation protein
EIM29759.1	nitrogen regulation protein NR(I)	PVE20899.1	nodulation protein NodU
EIM29496.1	nitrogen regulatory protein PII	PVE20900.1	nodulation factor ABC transporter
EIM29287.1	PTS IIA-like nitrogen-regulatory protein PtsN	PVE20901.1	nodulation protein NodJ
EIM29168.1	nitrogen regulatory protein PII	PVE20902.1	nodulation protein
EIM30624.1	ATP-binding ABC transporter family nodulation protein NodI	PVE20904.1	nodulation protein NodZ
EIM30627.1	Nodulation protein A (NodA)	PVE20614.1	nodulation protein NodF
